# NBPF1, a tumor suppressor candidate in neuroblastoma, exerts growth inhibitory effects by inducing a G1 cell cycle arrest

**DOI:** 10.1186/s12885-015-1408-5

**Published:** 2015-05-10

**Authors:** Vanessa Andries, Karl Vandepoele, Katrien Staes, Geert Berx, Pieter Bogaert, Gert Van Isterdael, Daisy Ginneberge, Eef Parthoens, Jonathan Vandenbussche, Kris Gevaert, Frans van Roy

**Affiliations:** 1 Inflammation Research Center, VIB, Ghent, Belgium; 2 Department of Biomedical Molecular Biology, Ghent University, Technologiepark 927, B-9052 Ghent, Zwijnaarde Belgium; 3 Department of Medical Protein Research, VIB, Ghent, Belgium; 4 Department of Biochemistry, Ghent University, Ghent, Belgium; 5 Laboratory for Molecular Diagnostics - Hematology, Ghent University Hospital, Ghent, Belgium; 6 BARC Global Central Laboratory, Ghent, Zwijnaarde Belgium; 7 Department of Internal Medicine, Ghent University, Ghent, Belgium; 8 BioImaging Core, VIB, Ghent, Belgium

**Keywords:** NBPF, Cell cycle, G1 arrest, p53, p21, Tumor suppressor, Cancer

## Abstract

**Background:**

*NBPF1* (Neuroblastoma Breakpoint Family, member 1) was originally identified in a neuroblastoma patient on the basis of its disruption by a chromosomal translocation t(1;17)(p36.2;q11.2). Considering this genetic defect and the frequent genomic alterations of the *NBPF1* locus in several cancer types, we hypothesized that *NBPF1* is a tumor suppressor. Decreased expression of *NBPF1* in neuroblastoma cell lines with loss of 1p36 heterozygosity and the marked decrease of anchorage-independent clonal growth of DLD1 colorectal carcinoma cells with induced *NBPF1* expression further suggest that NBPF1 functions as tumor suppressor. However, little is known about the mechanisms involved.

**Methods:**

Expression of NBPF was analyzed in human skin and human cervix by immunohistochemistry. The effects of NBPF1 on the cell cycle were evaluated by flow cytometry. We investigated by real-time quantitative RT-PCR the expression profile of a panel of genes important in cell cycle regulation. Protein levels of *CDKN1A*-encoded p21^CIP1/WAF1^ were determined by western blotting and the importance of p53 was shown by immunofluorescence and by a loss-of-function approach. LC-MS/MS analysis was used to investigate the proteome of DLD1 colon cancer cells with induced NBPF1 expression. Possible biological interactions between the differentially regulated proteins were investigated with the Ingenuity Pathway Analysis tool.

**Results:**

We show that NBPF is expressed in the non-proliferative suprabasal layers of squamous stratified epithelia of human skin and cervix. Forced expression of NBPF1 in HEK293T cells resulted in a G1 cell cycle arrest that was accompanied by upregulation of the cyclin-dependent kinase inhibitor p21^CIP1/WAF1^ in a p53-dependent manner. Additionally, forced expression of NBPF1 in two p53-mutant neuroblastoma cell lines also resulted in a G1 cell cycle arrest and *CDKN1A* upregulation. However, *CDKN1A* upregulation by NBPF1 was not observed in the DLD1 cells, which demonstrates that NBPF1 exerts cell-specific effects. In addition, proteome analysis of NBPF1-overexpressing DLD1 cells identified 32 differentially expressed proteins, of which several are implicated in carcinogenesis.

**Conclusions:**

We demonstrated that NBPF1 exerts different tumor suppressive effects, depending on the cell line analyzed, and provide new clues into the molecular mechanism of the enigmatic NBPF proteins.

**Electronic supplementary material:**

The online version of this article (doi:10.1186/s12885-015-1408-5) contains supplementary material, which is available to authorized users.

## Background

We originally identified human *NBPF1* (Neuroblastoma BreakPoint Family, member 1) in a neuroblastoma (NB) patient on the basis of its disruption in a *de novo*, constitutional translocation between chromosomes 1p36.2 and 17q11.2 [1-3]. *NBPF1* is a member of a gene family with intricate genomic structure [4]. The *NBPF* members are primarily located on duplicated regions of chromosome 1, and analysis of the predicted protein sequences showed that several pairs of exon types encode a protein domain called the NBPF/DUF1220 repeat [4, 5]. The number of encoded NBPF/DUF1220 repeats varies from 4 to 52 copies, depending on the gene member and the NBPF1 protein has 7 repeats [6]. The copy number of the NBPF/DUF1220 repeat is much larger in humans than in other primates, which suggests an important role in human evolution [4, 5]. The *NBPF* genes are likely involved in cancer and in brain and developmental disorders (reviewed in [7]). This has been ascribed to their location in unstable high-identity duplication blocks, which leads to recurrent chromosomal rearrangements. One tumor type of particular interest is NB.

NB tumors are derived from the sympathetic nervous system and account for approximately 15% of cancer deaths in children [8]. However, a fascinating feature of NB is its remarkable biological heterogeneity, which is evident in the broad variety of clinical courses of the disease. While some patients experience spontaneous regression or differentiation of the tumor, others are affected by rapid and fatal tumor progression despite increasingly intensive treatment strategies [9].

Evidence for the involvement of *NBPF* genes in NB comes from the abovementioned disruption of *NBPF1* in a NB patient, and from the association of NB with copy number variation of an *NBPF1* paralog [10]. Interestingly, the 1p36 region is frequently deleted not only in NB, but also in other human cancer types, including those of neural, epithelial and hematopoietic origin, indicating that the same tumor suppressor genes might be involved in a broad range of human cancers [11].

Based on these findings, we hypothesized that NBPF1 acts as a tumor suppressor. Previously, we reported that expression of *NBPF1* mRNA is significantly decreased in NB cell lines with loss of heterozygosity for 1p36 compared to cell lines with a normal 1p36 locus [3]. This decreased expression is a hallmark of tumor suppression activity. Moreover, NBPF1-expressing colon cancer cells formed significantly fewer colonies in soft agar than control cell lines, indicating that NBPF1 might be important for suppression of anchorage-independent growth [3].

In this study, we show that NBPF is expressed in the non-proliferative suprabasal layers of squamous stratified epithelia of human skin and cervix. Moreover, we show that forced expression of NBPF1 in the human HEK293T cell line resulted in a p53-dependent G1 cell cycle arrest that was accompanied by upregulation of the cyclin-dependent kinase inhibitor p21^CIP1/WAF1^. Additionally, overexpression of *NBPF1* in two p53-mutant NB cell lines resulted in G1 cell cycle arrest and concomitant *CDKN1A* induction. However, G1 cell cycle arrest and *CDKN1A* upregulation were not observed in a colon cancer cell line in which NBPF1 expression was induced, despite the clear NBPF1-dependent inhibition of anchorage-independent clonal growth in this cell line [3]. This demonstrates that NBPF1 exerts cell-specific tumor suppressive effects. In conclusion, this study advances the understanding of the role of NBPF1 as a tumor suppressor.

## Methods

### Reagents

Doxycycline hydrochloride (dox; Sigma, Bornem, Belgium) was used at a final concentration of 2 μg/ml to induce expression of NBPF1-IRES-EGFP or EGFP in DLD1Tr21/NBPF1 and DLD1Tr21/Mock cells, respectively. Doxorubicine hydrochloride (Doxo; Sigma) was used at a final concentration of 350 nM during 24 h. Aphidicolin (Sigma) was used at a final concentration of 2 μg/ml.

### Cell culture and transfections

Human HEK293tsA1609neo (in short HEK293T) and its derivative HEK_shRNAp53 (in which the expression of p53 has been silenced by an shRNA) were cultured in DMEM medium supplemented with 10% fetal calf serum (FCS), 2 mM glutamine and 0.4 mM sodium pyruvate. SH-SY5Y neuroblastoma cells (ATCC, CRL-2266) were cultured in DMEM medium supplemented with 15% FCS, 2 mM glutamine and 0.4 mM sodium pyruvate. The SK-N-AS (ATCC, CRL-2137) and NLF [12] neuroblastoma cell lines were cultured in RPMI medium supplemented with 10 % FCS, 2 mM glutamine and 0.4 mM sodium pyruvate. HEK293T cells were transfected using the calcium phosphate precipitation method, and neuroblastoma cells were transfected with Fugene HD (Roche Applied Science, Vilvoorde, Belgium). DLD1Tr21/Mock and DLD1Tr21/NBPF1 cells have been described and were grown in standard medium composed of RPMI with 10% FCS [3].

### Plasmid constructions

To construct the pdEGFP-NBPF1 plasmid for expression of EGFP-fused NBPF1 under the control of a CMV promoter, full-length NBPF1 cDNA was transferred to the pdEGFP vector by Gateway technology (Life Technologies Europe, Ghent, Belgium). The vector expressing EGFP-luciferase under the control of an SV40 promoter (pGL3-EGFP-luciferase) was a kind gift from Dr. Eric Raspé. To generate DLD1Tr21/NBPF1 cells, we cloned cDNA of NBPF1-IRES-EGFP, fused to an amino-terminal Flag tag, into the pcDNA4/TO vector (Life Technologies Europe). This plasmid expresses flag-tagged NBPF1, under the control of a doxycycline-inducible promoter [3].

### Immunofluorescence

Cells on glass coverslips were rinsed briefly with PBS and fixed with 4% paraformaldehyde. They were incubated for 1 h with primary antibody diluted in blocking solution (0.4% gelatin). Antibodies used were goat anti-NBPF sc-82241 (Santa Cruz Biotechnology, Heidelberg, Germany, dilution 1/200) and mouse anti-p53 DO-1 (Santa Cruz Biotechnology, dilution 1/250). Cells were then washed in PBS and incubated for 1 h with secondary antibodies coupled to Alexa fluorophores (Life Technologies Europe) diluted in blocking solution. Coverslips were mounted in Vectashield containing DAPI (Vector Laboratories, Peter Borough, United Kingdom) to prevent photobleaching.

### SDS-PAGE and immunoblotting

EGFP-positive and EGFP-negative populations of HEK293T, HEK293T_shRNAp53, SH-SY5Y, NLF and SK-N-AS cells were isolated at 48 h after transfection by FACS (Epics Altra, Beckman Coulter, California, USA) and used for protein extraction with Trizol (Life Technologies Europe). For subsequent SDS-PAGE analysis we used a 10 % gel and loaded a total of 40 μg protein per lane. After blotting to PVDF membranes (Millipore, Billerica, MA, USA), blocking with 5 % non-fat dry milk occurred. The membrane was washed 3 times with 5% w/v BSA, 1x TBS, 0.1% Tween® 20 and then incubated overnight with primary anti-p21^CIP1/WAF1^ antibody (rabbit anti-p21^CIP1/WAF1^ 12D1, Cell Signaling Technology, Beverly, MA, USA; dilution 1/1000) in the same buffer at 4 °C. After several washings, the membrane was incubated for 1 h with secondary horseradish peroxidase (HRP)-conjugated antibodies and proteins were detected by the enhanced chemiluminescence (ECL) detection system (GE Healthcare Europe, Diegem, Belgium). Anti-vinculin (mouse anti-vinculin, Sigma, dilution 1/1000) or anti-actin (mouse anti-actin, MP Biomedicals, dilution 1/10.000) detection was used as a loading control and anti-EGFP (rabbit anti-GFP A11122, Life Technologies, dilution 1/1000) or anti-NBPF detection (sc-82241, dilution 1/1000) was used to confirm transfection efficiency or in cell sorting experiments.

### Immunohistochemistry on paraffin-embedded tissue sections

Formalin-fixed, paraffin-embedded tissue specimens were retrieved from the archives of the Pathology Department at the University Hospital of Liège (protocol approved by the Liège University Hospital Ethics Committee). No other ethical approval was required for this study. Human and mouse tissues were fixed in 4% paraformaldehyde, dehydrated and paraffin-embedded. For staining, paraffin was removed and sections were rehydrated. Endogenous peroxidase was blocked with a 1/100 dilution of H_2_O_2_ in methanol. Antigen retrieval was carried out in a pressure cooker (2100 Retriever, PickCell Laboratories, Amsterdam, The Netherlands) with the sections soaked in citrate retriever buffer. Aspecific binding sites were blocked with 3 % horse serum in PBS + 1% BSA. Incubation with primary antibody (sc-82241, diluted 1/200 in PBS/1 % BSA) was done overnight at 4°C. Incubation with biotinylated secondary antibody (Dako, Glostrup, Denmark, dilution 1/300) was carried out for 30 min at room temperature. This was followed by a 20-min incubation with StreptABComplex/HRP (Dako) according to the manufacturer’s protocol. After washing, the sections were treated with DAB (BioGenex, Fremont, CA, USA) and counterstained with hematoxylin following standard procedures. Finally, the sections were embedded in Pertex (HistoLab, Gothenburg, Sweden).

### Time-lapse microscopy

HEK293T cells were transfected in an eight-well LabTek II chamber glass slide (Sigma) with plasmids encoding either EGFP-NBPF1 or EGFP-luciferase. After 6 h, the transfection medium was replaced with fresh medium and in one set of experiments aphidicolin was added after 24 h. At 48 h after transfection, EGFP-positive cells were selected for time-lapse recording using a cell observer spinning disk system (Zeiss, Zaventem, Belgium). This system includes an observer Z.1 microscope equipped with a Yokogawa disk CSU-X1. Images were taken with a pln Apo 40x/1.4 oil DIC III objective and a Rolera em-c_2_ Camera. The setup includes an incubation chamber, maintained at 37°C in a 5% CO_2_ atmosphere. DIC and fluorescence images were obtained every 10 min for 15 h. Images were processed into movies using Fiji (Just ImageJ; http://pacific.mpi-cbg.de/).

### Cell cycle analysis

Cells were harvested 48 h after transfection and fixed for 1 h in ice-cold ethanol. Fixed cells were treated with RNase A (Sigma, 1 mg/ml) and stained with propidium iodide (Sigma, 50 μg/ml). The cellular DNA content of EGFP-positive and EGFP-negative populations was evaluated using flow cytometry (FACSVerse; Becton Dickinson, San Jose, California, USA). We applied software-based compensation (Flowjo) to the collected data. Compensation matrix was defined with reference to single color controls. Gating strategy: 1/ side scatter versus forward scatter to exclude debris; 2/ FSC-height versus FSC-area for doublet discrimination; 3/ EGFP (FITC) versus forward scatter to identify EGFP-negative and EGFP-positive subpopulations. Cells with very strong EGFP expression were not taken into account for cell cycle analysis because time lapse experiments had shown that cells with such strong EGFP-NBPF1 expression were dying, and so we considered these EGFP levels as not physiological. All experiments were performed at least three times, and one representative figure is shown.

### Real-time qRT-PCR

EGFP-positive and EGFP-negative populations of HEK293T and HEK293T_shRNAp53 cells were isolated at 48 h after transfection by FACS (Epics Altra, Beckman Coulter, California, USA) and used for RNA extraction and real-time qRT-PCR analysis. RNA was prepared from the different cell populations with Trizol (Life Technologies Europe). cDNA was prepared with the iScript cDNA synthesis kit according to the manufacturer's instructions (Bio-Rad). qRT-PCR mixes contained 20 ng template cDNA, SensiFast SYBR No-ROX kit (GC Biotech, Alphen aan den Rijn, The Netherlands) and 300 nM forward and reverse primers. Reactions were performed on a LightCycler 480 (Roche) using the following protocol: incubation at 95°C for 5 min, followed by 40 cycles: 95°C for 10 s, 60°C for 30 s, and 72 °C for 1 s. Primers used are listed in Table 1. Gene expression levels were normalized using the geometric mean of the most stable reference genes [13].

**Table 1:** List of primer sequences used for quantitative RT-PCR of the genes indicated

Gene	Forward (5' to 3')	Reverse (5' to 3')
ANAP2	GACAGGAGTTGTTAGTGGCCT	TCCAGGACCGAGTGTAGCC
ATM	ACGTTACATGAGCCAGCAAAT	GAAAATGAGGTGGATTAGGAGCA
ATR	TCTCAGCCAACCTCCGTGAT	GCAGAAGTCTCGTTATGATCCAA
CCND1	GTGCTGCGAAGTGGAAACC	ATCCAGGTGGCG ACGATCT
CCNE1	GAGCCAGCCTTGGGACAATAA	GCACGTTGAGTTTG GGTAAACC
CDC34	GACGAGGGCGATCTATACAACT	GAGTATGGGTAGTCGATGGGG
CDK2	GCTAGCAGACTTTGGACTAGCCAG	AGCTCGGTACCACAGGGTCA
CDK4	ATGTTGTCCGGCTGATGGA	CACCAGGGTTACCTTGATCTCC
CDK6	GCGCCTATGGGAAGGTGTTC	TTGGGGTGCTCGAAGGTCT
CDKN1A	CGCTAATGGCGGGCTG	CGGTGACAAAGTCGAAGTTCC
CDKN1B	CTGCAACCGACGATTCTTCTACT	GGGCGTCTGCTCCACAGA
CUL1	ACCAGTCAAACCAAGCACGAG	GTCTGCCCCTTTTTCGACTTAG
CUL2	ACGACAATAAAAGCCGTGGTC	GGTTCAGGATAGGCCACACATA
CUL3	TGTGGAGAACGTCTACAATTTGG	TGCGCCTCTGTCTACGACT
SKP2	ACCTCCAGGAGATTCCAGACC	CCCAGGTTTGAGAGCAGTTCC
TP53	CCTCAGCATCTTATCCGAGTGG	TGGATGGTGGTACAGTCAGAGC
NBPF1	GCGAGGCTGCCCGAGCTTCT	GACTTCGCGTAACTTCCCATTCA
EGFP	GAGCTGAAGGGCATCGACTT	TCTGCTTGTCGGCCATGAT

### Proteome analysis

DLD1Tr21/Mock and DLD1Tr21/NBPF1 cells were grown for 4 days in their standard medium, containing 2 μg/ml dox. Cell pellets were re-suspended in 0.5 ml of cell lysis buffer (50 mM sodium phosphate, pH 7.5, 100 mM NaCl, 0.8% (w/v) CHAPS, and Complete Protease Inhibitor cocktail (Roche)). The insoluble fraction was removed by centrifugation (15 min at 16.000 g at 4°C). Samples were desalted on a NAP5 column in 20 mM tri-ethylammonium bicarbonate (pH 7.0). Endoproteinase Lys-C digestion was carried out at 37°C overnight using a ratio of 1/100 (w/w) endoproteinase Lys-C/protein. Peptides from the DLD1Tr21/Mock cell lysate were post-metabolically labeled with light NHS-^12^C_3_-propionate, whereas the peptides from DLD1Tr21/NBPF1 cell lysate were labeled with heavy NHS-^13^C_3_-propionate. Equal amounts of the peptide mixtures were combined, concentrated by vacuum-drying and re-dissolved in 1% acetic acid. The peptide mixture was then separated by RP-HPLC chromatography and 20 fractions were collected for further LC-MS/MS analysis. LC-MS/MS analysis was done on a LTQ-Orbitrap mass spectrometer. We acquired 158,141 MS/MS spectra, from which we identified 13,315 unique peptides (Swiss-Prot database, restriction to human proteins) and 3613 proteins (Mascot software used at 99% confidence). Mascot Distiller was then used to quantify the proteins and only proteins with a minimum of two quantifiable peptides in the experiment were included in the Ingenuity Pathway Analysis (IPA, Ingenuity Systems, Redwood City, CA). Keratins and ribosomal proteins were considered non-specific and were excluded from IPA analysis.

## Results

### NBPF members are expressed in the suprabasal layers of squamous stratified epithelia

**Fig. 1:**
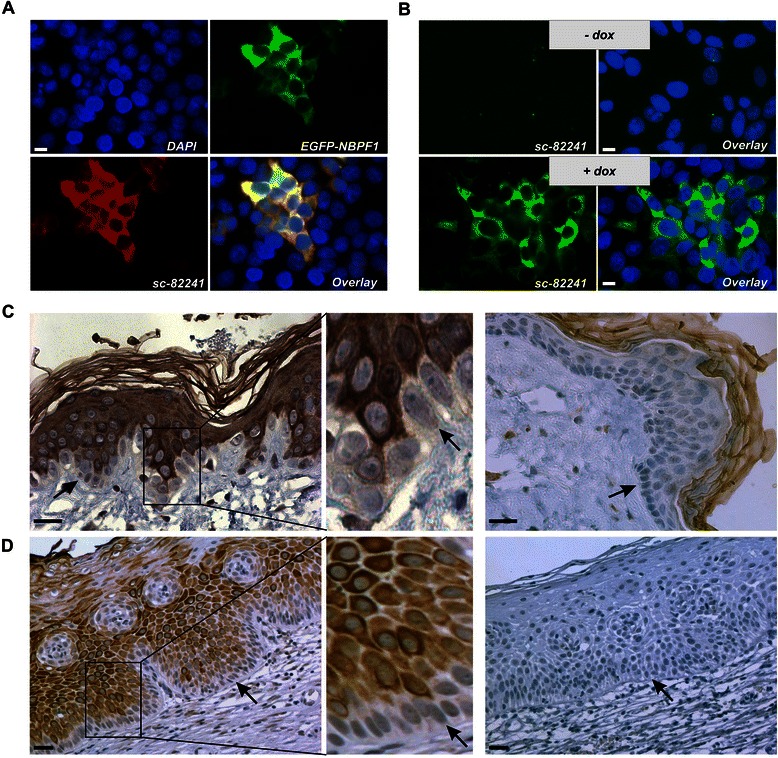
Polyclonal antibody sc-82241 recognizes NBPF proteins specifically and detects NBPF expression in suprabasal layers of normal human skin and cervix. (**A**) Upon overexpression of NBPF1, fused to EGFP, in HEK293T cells, a clear overlap is detected between the EGFP signal and the signal of the anti-NBPF antibody sc-82241. Nuclei were stained with DAPI. Scale bar: 10 μm. (**B**) The anti-NBPF antibody yields a specific cytoplasmic staining in DLD1Tr21/NBPF1 cells when NBPF1 expression is induced in the presence of dox. Scale bar: 10 μm. (**C**) Paraffin sections from human (left panel) and mouse skin (right panel) were stained with anti-NBPF antibody sc-82241. In the human epidermis, staining for NBPF is observed only in the non-proliferating, differentiated cells of the suprabasal layer and not in the proliferating basal cell layer (arrows). The middle panel shows a magnification of the boxed area. Mouse skin served as a negative control: no staining is seen in any of the living layers of the epidermis. The cornified envelope of the epidermis shows aspecific staining, since the signal is obtained in both mouse and human samples. Scale bars: 25 μm. (**D**) Normal human cervix stained with anti-NBPF antibody sc-82241 shows strong immunopositivity of the suprabasal layer. The middle panel shows a magnification of the boxed area. In the negative control (right panel), the primary antibody was omitted. Arrows point to basal cell layers. Scale bars: 25 μm

To explore the expression of the NBPF family members in more detail, we detected endogenous NBPF proteins in paraffin-embedded sections of human skin and cervix using a commercially available polyclonal anti-NBPF antibody (sc-82241). This antibody is raised against a peptide located in the protein domain encoded by exon types 5 and 1 (amino acids 240–290, UniProtKB Q3BBV1) and recognizes several NBPF proteins, including NBPF1. Because only few anti-NBPF antibodies are commercially available, it was important to confirm the specificity of this particular antibody. In immunofluorescence experiments, we used sc-82241 to stain HEK293T cells overexpressing an EGFP-fused NBPF1 protein: there was a clear overlap between the EGFP signal and sc-82241 reactivity (Fig. 1A). We then stained the inducible DLD1Tr21/NBPF1 stable cell line [3]: sc-82241 reactivity was observed only in the presence of doxycycline (dox), implying NBPF1-specific recognition (Fig. 1B). Taken together, these experiments clearly show that sc-82241 specifically detects human NBPF proteins.

Staining of human skin sections with the anti-NBPF antibody revealed a strong and specific staining of the suprabasal layer of the epidermis (Fig. 1C, left panel), in contrast to the proliferating basal layer [14, 15]. The mouse genome does not contain *NBPF* genes, and indeed we could not observe any staining in the living layers of mouse skin (Fig. 1C, right panel). We thus conclude that the observed staining in human skin is specific for NBPF. Additionally, we used this antibody to stain human cervix, another tissue with a squamous epithelium. Although the staining was less homogeneous here, we also found the strongest signal in the suprabasal layer in contrast to the proliferative basal layer (Fig. 1D).

### NBPF1 inhibits cell cycle progression in transfected human embryonic kidney cells

Tumor suppressor genes are vital for counteracting the formation and progression of cancers. Therefore, such genes generally function to slow down cell division, repair DNA errors or trigger apoptotic pathways [16]. We previously reported that NBPF1 induction suppresses anchorage-independent growth [3]. Moreover, the abovementioned expression of NBPF in suprabasal, non-proliferating layers of human skin and cervix pointed to an association between NBPF expression and inhibition of cell proliferation. This prompted us to explore the role of *NBPF1* expression during the cell cycle.

**Fig. 2:**
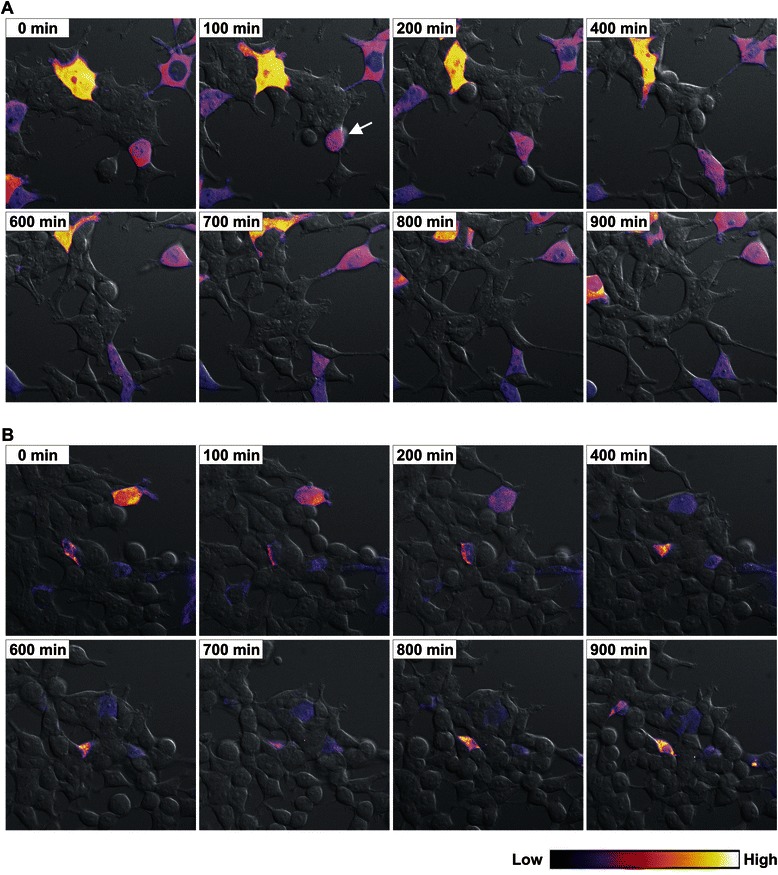
Time-lapse microscopy of NBPF1 expression in transfected HEK293T cells. Expression of EGFP-luciferase or EGFP-NBPF1 was followed for 15 h with images captured every 10 min. Individual frames are shown. Original movies are available as Additional files 1 and 2. Fluorescence levels are represented by pseudocolors, where blue indicates low EGFP levels and yellow indicates high EGFP levels (ImageJ LUT; color codes shown at the bottom). (**A**) Expression of EGFP-luciferase in cells remained at a constant level at all the indicated time points and did not interfere with cell division or induce cell death. The arrow points to an EGFP-positive mitotically dividing cell. (**B**) Expression of EGFP-NBPF1 in cells fluctuated, with some cells showing decreasing levels and others showing delayed expression. In no case were EGFP-NPBP1 expressing mitotic cells observed

**Fig. 3:**
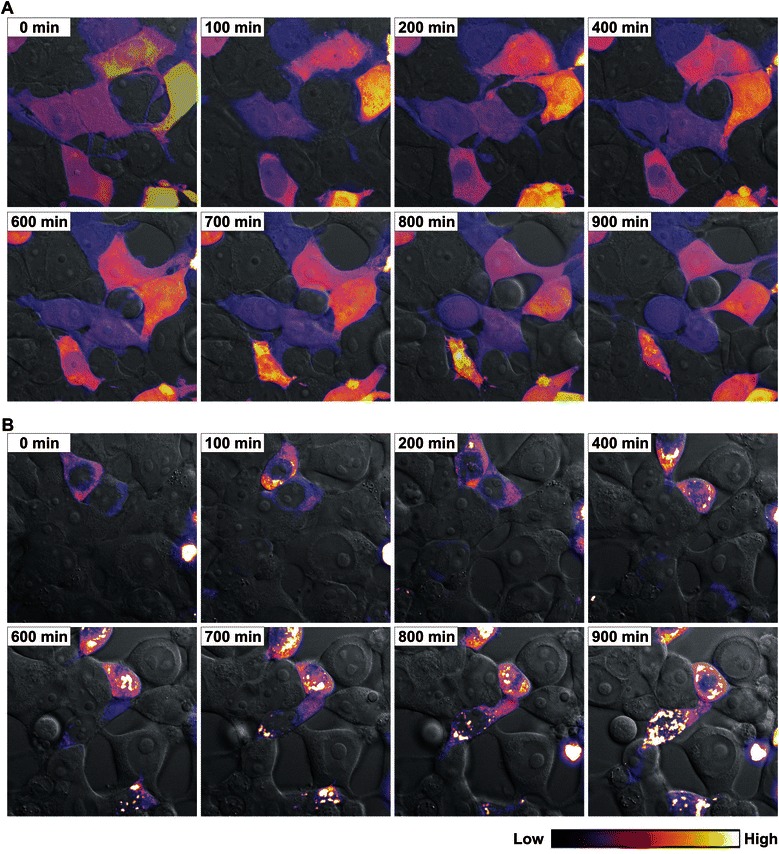
Time-lapse microscopy of NBPF1 expression in transfected HEK293T cells stimulated with aphidicolin. EGFP-luciferase or EGFP-NBPF1 expression was followed for 15 h with images captured every 10 min. Individual frames are shown. Original movies are available as Additional files 3 and 4. Fluorescence levels are represented by pseudocolors, with blue indicating low EGFP levels and yellow indicating high EGFP levels (ImageJ LUT; color codes shown at the bottom). (**A**) Expression of EGFP-luciferase in cells stimulated with aphidicolin remained at a constant level at all the indicated time points. (**B**) Expression of EGFP-NBPF1 in cells stimulated with aphidicolin showed increased accumulation in the form of particulate or aggregated structures. In no case were cells with decreasing levels of EGFP-NBPF1 observed

Therefore, we investigated the spatio-temporal dynamics of NBPF1 expression in the human embryonic kidney cell line, HEK293T. These cells were transfected with a control chimeric EGFP-luciferase expressing plasmid or with a plasmid expressing a chimeric EGFP-NBPF1 protein. EGFP fluorescence for both setups was followed in real-time. Interestingly, striking differences were seen between the control cells and the EGFP-NBPF1 positive cells. Whereas EGFP-luciferase expression remained at a constant level for at least 15 h (Fig. 2A and representative movie in Additional file 1: Movie 1), EGFP-NBPF1 expression often decreased rapidly (Fig. 2B and representative movie in Additional file 2: Movie 2). Also, we observed cells in the EGFP-NBPF1 setup that were EGFP negative at the onset of the experiment, but became EGFP positive later on. Importantly, we never observed any cell expressing EGFP-NBPF1 that completed a full cell cycle, whereas untransfected cells and EGFP-luciferase-transfected control cells divided frequently. Therefore, we hypothesized that the cell cycle was arrested in EGFP-NBPF1 positive cells, at least during the period of examination. Additionally, the disappearance of EGFP-NBPF1 positive cells suggested that the protein was degraded in proliferating cells. To further investigate this, we induced growth arrest by using a chemotherapeutic agent, aphidicolin, which is an inhibitor of eukaryotic nuclear DNA replication and blocks the cell cycle at the early S phase. HEK293T cells were transfected with an EGFP-luciferase control plasmid or with a plasmid expressing a chimeric EGFP-NBPF1 protein. After 24 h, the cells were treated with aphidicolin and EGFP fluorescence for both setups was followed in real-time 24 h later. Strikingly, EGFP-NBPF1 expression did not decrease but the protein accumulated in the form of particulate or aggregated structures during the time of imaging (Fig. 3B and representative movie in Additional file 3: Movie 3) whereas EGFP-luciferase expression remained at a constant level and no aggregates were seen (Fig. 3A and representative movie in Additional file 4: Movie 4). Therefore, we hypothesized that NBPF1 expression is indeed lost in proliferating cells, and that upon artificial induction of a cell cycle block, NBPF1 escapes degradation but loses its functionality due to its aggregation. Taken together, these data indicate that NBPF1 overexpression affects cell cycle and cell fate, and reveal that NBPF1 expression is cell cycle dependent: expression occurs mainly in G1 and is lost during mitosis.

**Fig. 4:**
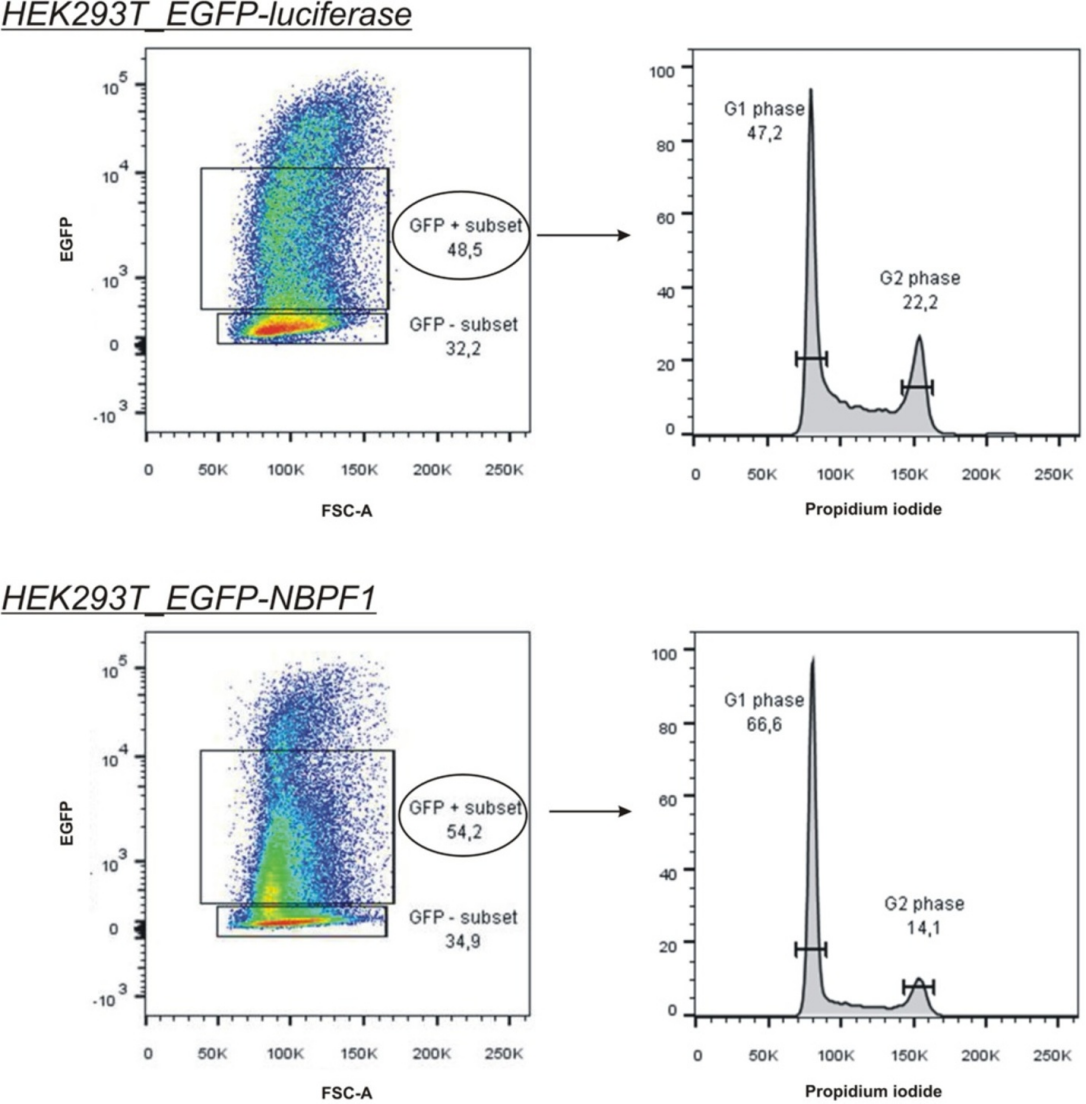
NBPF1 inhibits cell cycle progression in transfected HEK293T cells. HEK293T cells were transfected with plasmids encoding either EGFP-luciferase (negative control) or EGFP-NBPF1. Cell cycle profiles were determined 48 h after transfection by flow cytometry on the effectively transfected (EGFP-positive) cell subpopulations. Transfection efficiency is represented as percentages in the left panels. The different cell cycle phases are shown only for the effectively transfected (EGFP-positive) subpopulations (right panels). Cells in G1 phase (2n DNA content) and cells in G2 phase (4n DNA content) are annotated on the histograms and calculated as percentage of the whole population

**Fig. 5:**
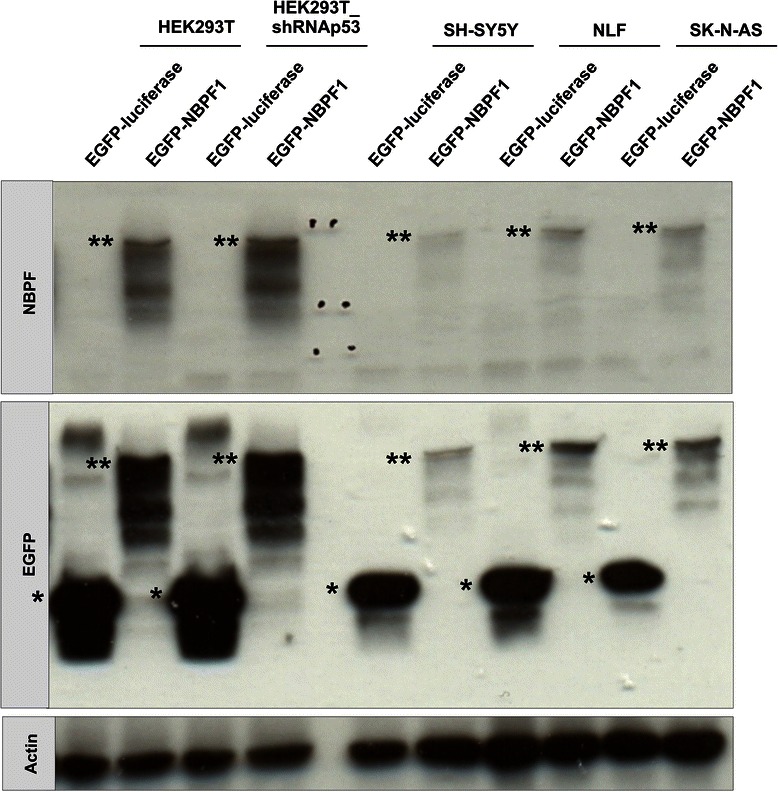
EGFP-luciferase and EGFP-NBPF1 expression in the cell lines used for flow cytometry in this study. Different cell lines (HEK293T, HEK293T_shRNAp53, SH-SY5Y, NLF and SK-N-AS) were transfected with plasmids expressing either EGFP-luciferase or EGFP-NBPF1 in order to investigate cell cycle distribution. As a control for efficient transfection and expression of EGFP-luciferase or EGFP-NBPF1, we took a fraction of the cells and prepared equal amounts of total cellular lysates for SDS-PAGE. These lysates were immunoblotted with an anti-NBPF antibody (sc-82241) to detect overexpressed NBPF1 protein, with an anti-EGFP antibody to detect the fusion proteins EGFP-luciferase (indicated by *) and EGFP-NBPF1 (indicated by **), or with an anti-actin antibody, which served as a loading control. All cell lines transfected for flow cytometry analysis showed effective expression of EGFP-luciferase or EGFP-NBPF1 proteins

Further, we examined the effects of NBPF1 overexpression on cell cycle progression by flow cytometry. We transiently overexpressed either EGFP-luciferase or EGFP-NBPF1 in HEK293T cells and 48 h after transfection, cells were fixed, stained with propidium iodide and cell cycle profiles were examined for successfully transfected cells (Fig. 4, left panels; 48.5% EGFP-positive cells for EGFP-luciferase expressing cells; 54.2% for EGFP-NBPF1 expressing cells). This experiment clearly demonstrated that EGFP-NBPF1 overexpression blocked cells in the G1 phase of the cell cycle (Fig. 4, right panels). As a control for efficient EGFP-NBPF1 expression we determined the protein levels of NBPF1 and confirmed that, as expected, only cells transfected with EGFP-NBPF1 showed strong NBPF1 expression, whereas cells transfected with EGFP-luciferase did not (Fig. 5).

### NBPF1 inhibits cell cycle progression in HEK293T cells by increasing p21^CIP1/WAF1^ levels in a p53-dependent manner

**Fig. 6:**
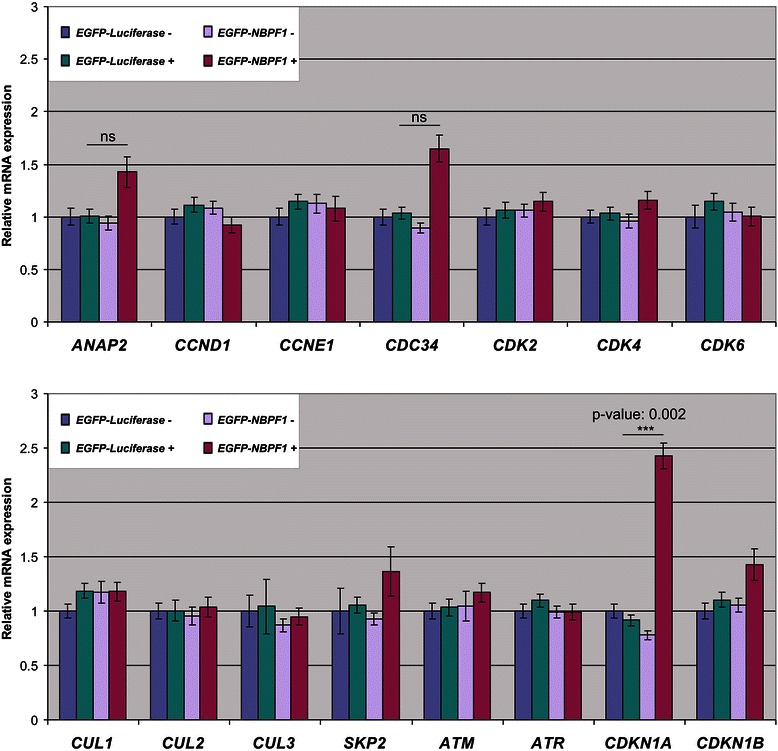
NBPF1 overexpression increases *CDKN1A* transcript levels. HEK293T cells were transfected with plasmids encoding either EGFP-luciferase or EGFP-NBPF1. Both EGFP-positive (+) and EGFP-negative populations (−) were isolated by FACS, and the expression levels of important cell cycle related genes were determined by real-time qRT-PCR. The values of EGFP-luciferase negative cells were set to 1. p-values were calculated with one-way ANOVA; ns: not significant

To gain more insight into the mechanism of the G1 arrest upon NBPF1 expression, we assessed the expression of several genes known to be important in the cell cycle. HEK293T cells were transfected with plasmids encoding either EGFP-luciferase or EGFP-NBPF1. 48 hours later, EGFP-positive and EGFP-negative populations from both settings were isolated by FACS (fluorescence-activated cell sorting). For all populations, we investigated by real-time quantitative RT-PCR (qRT-PCR) the expression profile of a panel of relevant genes. We focused on genes that are master regulators of the G1 phase or the G1-to-S transition (*ANAP2, CCND1, CCNE1, CDC34, CDK2, CDK4, CDK6, CUL1, CUL2, CUL3, SKP2*), and on genes important in cell cycle checkpoints (*ATM, ATR*) and cell cycle arrest (*CDKN1A, CDKN1B*). This profiling showed that expression of NBPF1 led to a reproducible 2.5-fold increase in *CDKN1A* mRNA, which encodes the cell cycle inhibitor p21^CIP1/WAF1^ (Fig. 6). This increase was seen only in EGFP-NBPF1-positive cells and not in the control or mock transfected cells. The profiling also showed a modest NBPF1-induced upregulation of the mRNA levels of *ANAP2, CDC34* and *CDKN1B*. However, in contrast to the obvious and reproducible *CDKN1A* induction, the upregulation of these three genes turned out to be poorly reproducible (data not shown). As a control for efficient transfection and cell sorting, we determined the mRNA expression levels of NBPF1 and confirmed that, as expected, only the EGFP-positive population of cells transfected with EGFP-NBPF1 showed strong NBPF1 expression, whereas the EGFP-negative populations and both populations transfected with EGFP-luciferase did not (data not shown).

**Fig. 7:**
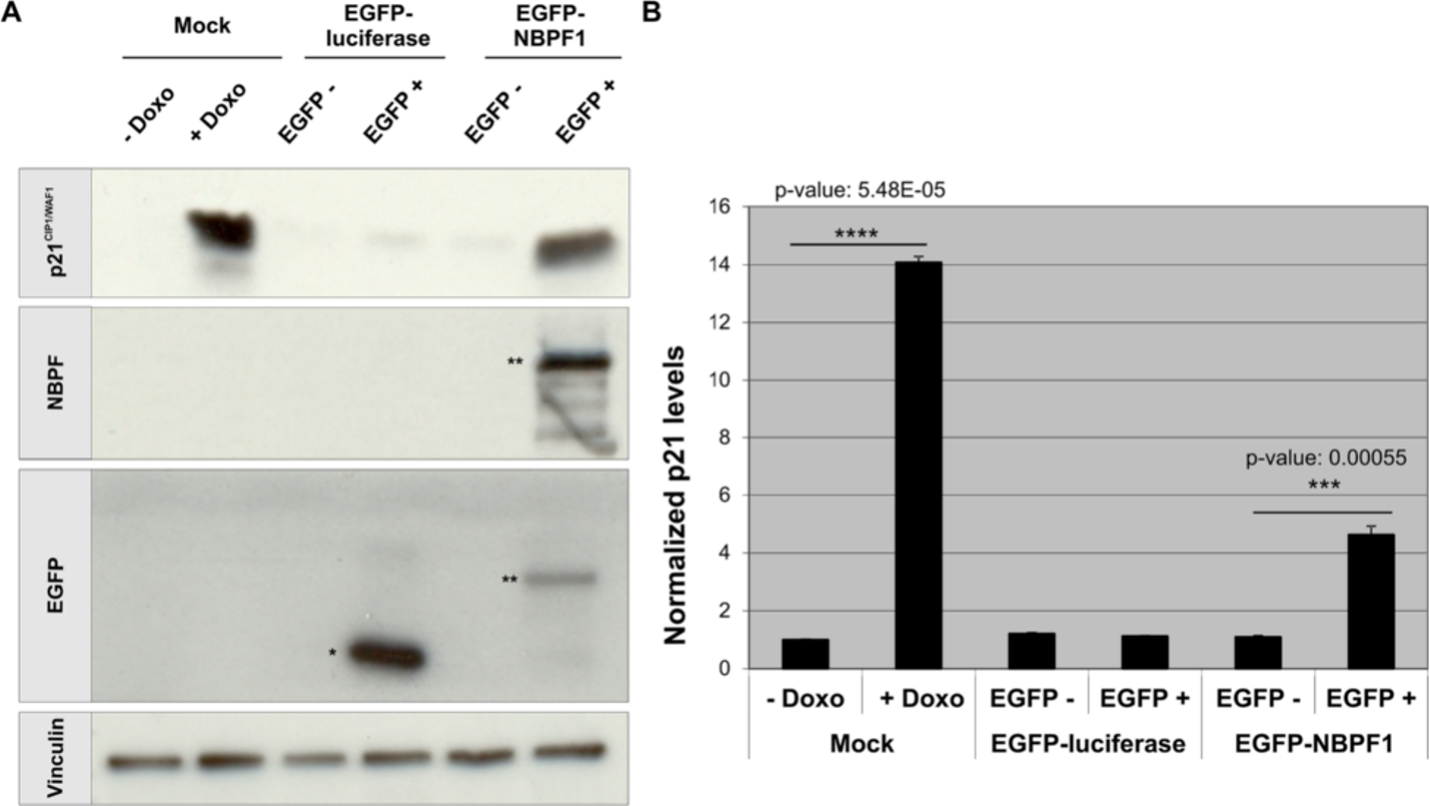
NBPF1 overexpression increases p21^CIP1/WAF1^ protein levels. (**A**) HEK293T cells were transfected with plasmids encoding either EGFP-luciferase or EGFP-NBPF1. Both EGFP-positive (+) and EGFP-negative populations (−) were isolated by FACS, and the protein levels of p21^*CIP1/WAF1*^ were detected by western blotting (top row). Treatment of cells with doxorubicin (+ Doxo) served as a positive control for p21 induction. Detection with the anti-NBPF antibody (sc-82241) showed the successful isolation of EGFP-NBPF1 transfected cells (second row). Detection with an anti-EGFP antibody (third row) showed successful isolation of transfected cells that were EGFP-luciferase positive (indicated by *) or EGFP-NBPF1 positive (indicated by **). Vinculin expression acted as a loading control (bottom row). (**B**) Quantification of p21^CIP1/WAF1^ signals in the blot of (A), normalized against vinculin signals. In addition to the positive control (+ Doxo), only cells expressing EGFP-NBPF1 showed clear induction of p21. p-values were calculated with one-way ANOVA

In addition, protein lysates of EGFP-positive and EGFP-negative populations from both settings showed that not only the *CDKN1A* mRNA levels, but also the p21^CIP1/WAF1^ protein levels were upregulated upon NBPF1 overexpression in HEK293T cells (Fig. 7). Doxorubicin (Doxo) treatment of mock transfected cells served as a positive control for p21^CIP1/WAF1^ induction. Taken together, these data indicated that NBPF1 overexpression in HEK293T cells induces p21^CIP1/WAF1^ expression at both the mRNA and protein levels.

**Fig. 8:**
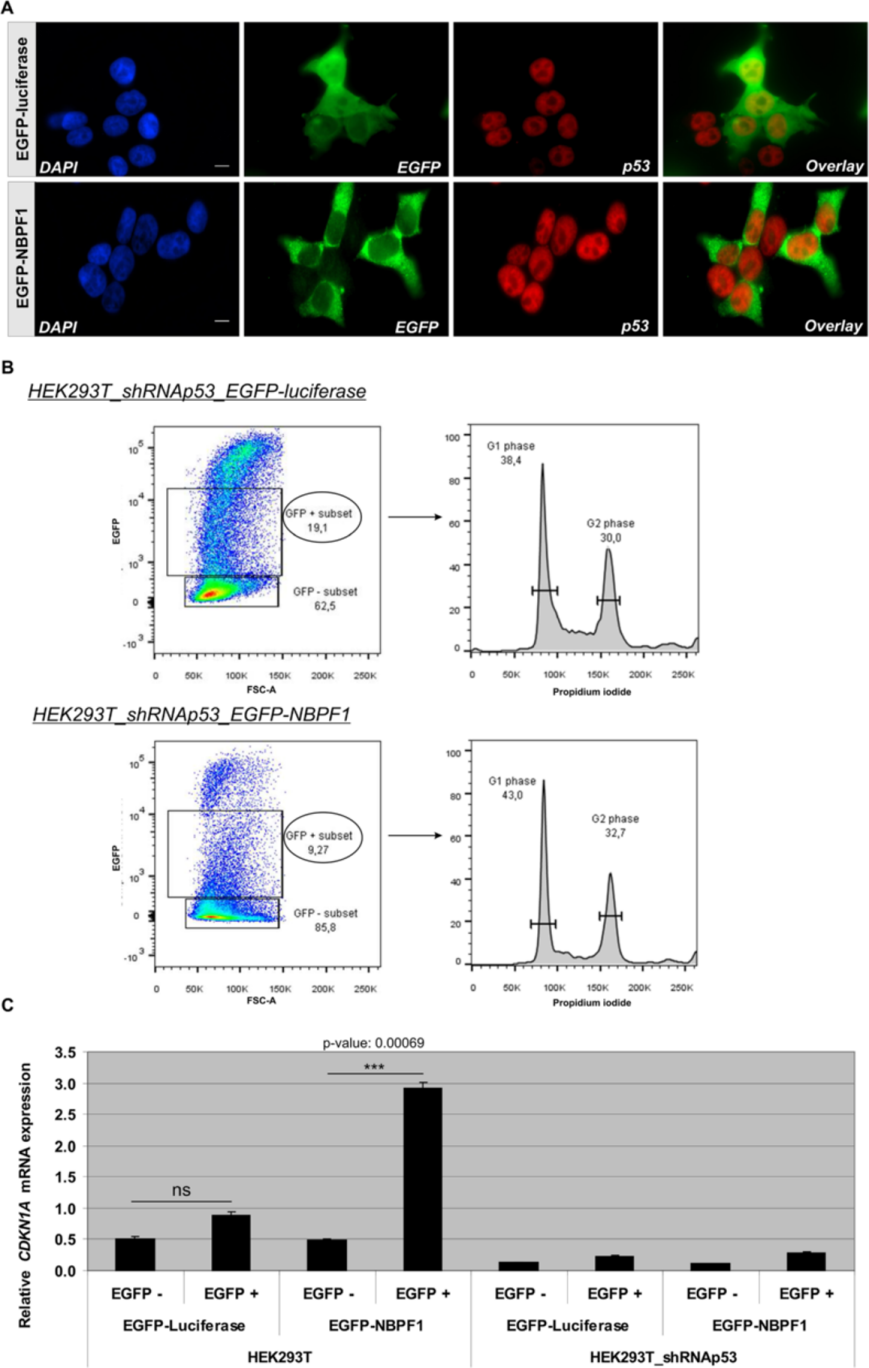
NBPF1 overexpression induces nuclear accumulation of p53 as well as p53-dependent cell cycle arrest and *CDKN1A* induction. (**A**) HEK293T cells were transfected with a plasmid encoding either EGFP-luciferase (top panels) or EGFP-NBPF1 (lower panels). Immunofluorescence at 48 h after transfection for p53 revealed an increased accumulation in the nucleus upon EGFP-NBPF1 transfection. Nuclei were counterstained with DAPI. Scale bars: 10 μm. (**B**) HEK293T_shRNAp53 cells were transfected with plasmids encoding either EGFP-luciferase or EGFP-NBPF1. Cell cycle profiles were determined 48 h after transfection by flow cytometry on the effectively transfected (EGFP-positive) cell subpopulations. Transfection efficiency is represented as percentage in the left panels. The different cell cycle phases are shown only for the effectively transfected (EGFP-positive) subpopulations (right panels). Cells in G1 phase (2n DNA content) and cells in G2 phase (4n content) are annotated on the histograms and calculated as percentage of the whole population. NBPF1 overexpression does not induce G1 cell cycle arrest in HEK293T cells with stable knockdown of p53. (**C**) HEK293T and HEK293T_shRNAp53 cells were transfected with plasmids encoding either EGFP-luciferase or EGFP-NBPF1. Both EGFP-positive (+) and EGFP-negative populations (−) were isolated by FACS, and the expression levels of *CDKN1A* transcripts were determined by real-time qRT-PCR. EGFP-NBPF1 expression induced *CDKN1A* transcripts only in HEK293T cells. p-values were calculated with one-way ANOVA; ns: not significant

To examine whether the upregulation of *CDKN1A* upon NBPF1 expression was p53-dependent, we analyzed p53 expression in HEK293T cells transfected with plasmids encoding either EGFP-luciferase or EGFP-NBPF1. 48 hours later, we performed immunofluorescence for EGFP and p53 and observed that the overexpression of NBPF1 resulted in increased nuclear accumulation of p53 in comparison with the EGFP-luciferase transfected control cells (Fig. 8A). To confirm the role of p53 by a different approach, we used HEK293T cells in which p53 had been stably silenced (HEK293T_shRNAp53). We first investigated the different cell cycle phases upon overexpression of EGFP-luciferase or EGFP-NBPF1. This experiment showed that in HEK293T cells with stable knockdown of p53, NBPF1 overexpression does not result in a G1 cell cycle arrest (Fig. 8B). As a control for efficient EGFP-NBPF1 expression, we determined the protein levels of NBPF1 and confirmed that, as expected, only cells transfected with EGFP-NBPF1 showed strong NBPF1 expression, whereas cells transfected with EGFP-luciferase did not (Fig. 5).

**Fig. 9:**
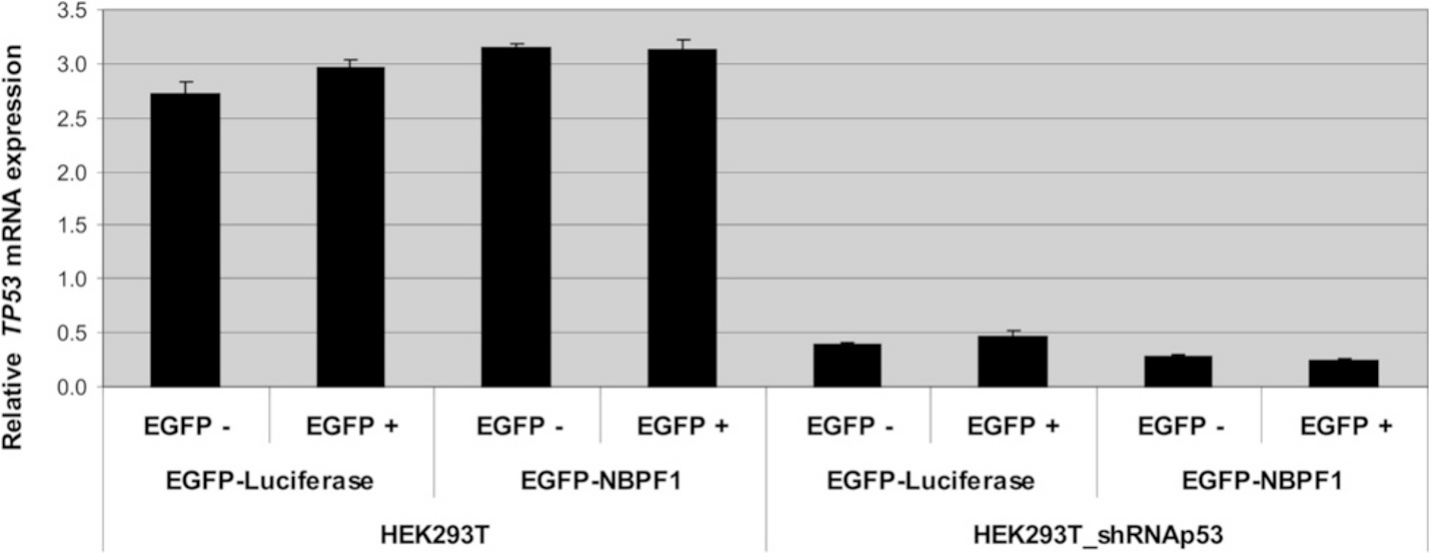
NBPF1 overexpression has no effect on the mRNA levels of the *TP53* gene. HEK293T and HEK293_shRNAp53 cells were transfected with plasmids encoding either EGFP-luciferase or EGFP-NBPF1. Both EGFP-positive (+) and EGFP-negative populations (−) were isolated by FACS and the expression levels of *TP53* were determined by real-time quantitative RT-PCR

In addition, we investigated whether overexpression of NBPF1 in this modified cell line still results in the upregulation of *CDKN1A* mRNA levels. HEK293T (no silencing of p53) and HEK293T_shRNAp53 cells were transfected with plasmids encoding either EGFP-luciferase or EGFP-NBPF1. EGFP-positive and EGFP-negative populations from both settings were isolated by FACS, and then mRNA expression levels of *CDKN1A* were investigated by real-time qRT-PCR in all populations. Whereas *NBPF1* overexpression in wild type cells resulted in upregulation of *CDKN1A* mRNA levels, as initially observed, this was not the case when p53 had been silenced (Fig. 8C). Additionally, NBPF1 overexpression did not influence *TP53* mRNA levels, neither in HEK293T nor in HEK293T_shRNAp53 cells (Fig. 9).

Together, these data indicate that overexpression of NBPF1 in HEK293T cells results in a p53-dependent induction of *CDKN1A* and cell cycle arrest.

### NBPF1 overexpression in neuroblastoma cell lines

**Fig. 10:**
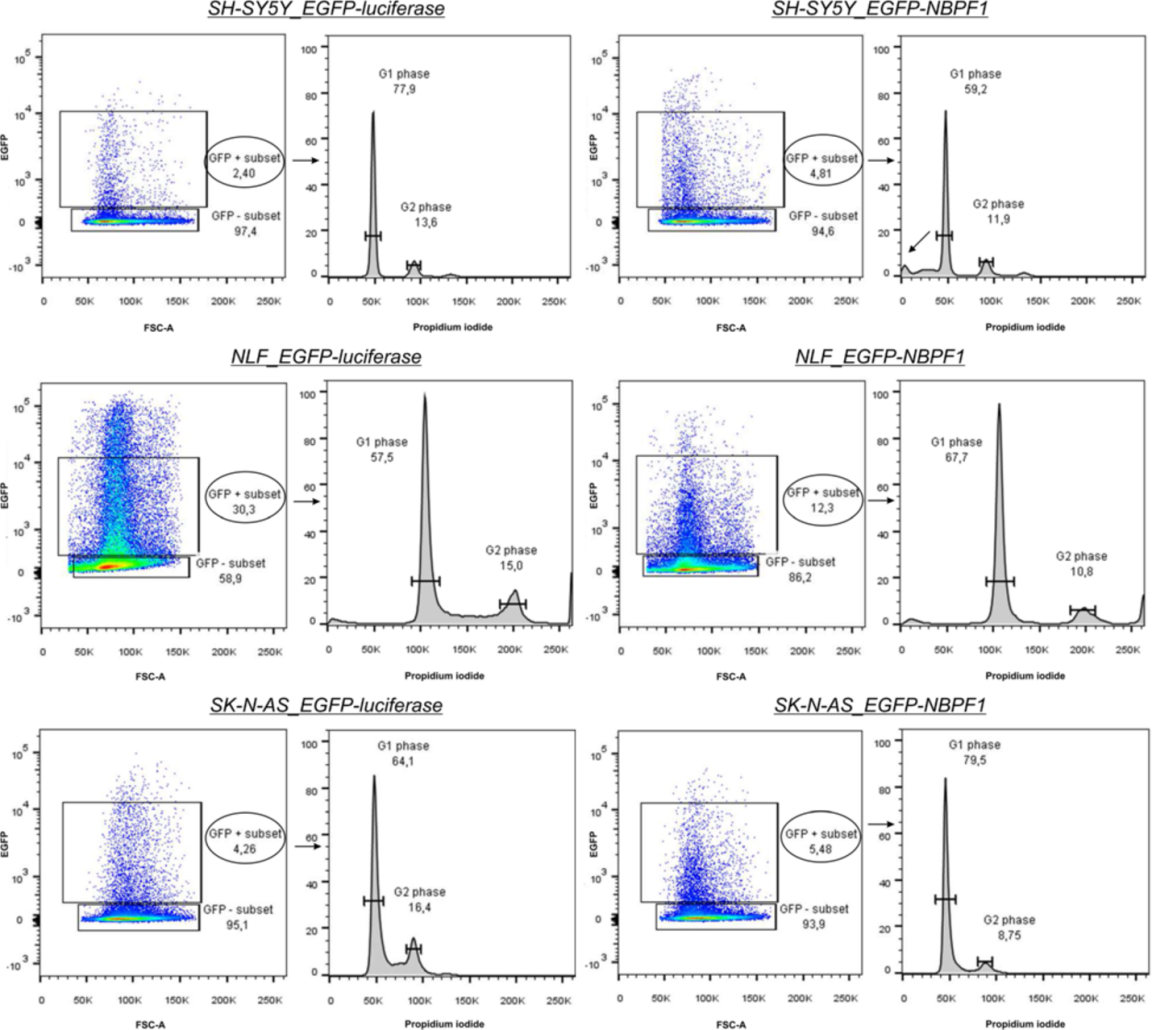
NBPF1 induces cell death in an NB cell line with wild-type p53 and inhibits cell cycle progression in NB cell lines with mutated p53. Three different NB cell lines were transfected with plasmids encoding either EGFP-luciferase (left panels) or EGFP-NBPF1 (right panels) and cell cycle profiles were analyzed 48 h post-transfection. Transfection efficiency is represented as percentage in the left panels. The different cell cycle phases are shown only for the effectively transfected (EGFP-positive) subpopulations (right panels). Cells in G1 phase (2n DNA content) and cells in G2 phase (4n content) are annotated on the histograms and calculated as percentage of the whole population. Overexpression of NBPF1 in SH-SY5Y cells, bearing a wild-type *TP53* gene, does not result in G1 cell cycle arrest, but induces cell death (sub-G1 peak, indicated by arrow) (top panel). Overexpression of NBPF1 in two NB cell lines, NLF (middle panel) and SK-N-AS (bottom panel), with mutated p53 induces G1 cell cycle arrest

The human *NBPF1* gene was originally identified in a NB patient on the basis of its disruption in a *de novo*, constitutional translocation between chromosomes 1p36.2 and 17q11.2 [1-3]. Moreover, we reported that expression of *NBPF1* mRNA is significantly decreased in NB cell lines with loss of heterozygosity for 1p36 compared to cell lines with a normal 1p36 locus [3]. This decreased expression is a hallmark of tumor suppression activity, which motivated us to investigate whether NBPF1 overexpression arrests the cell cycle also in neuroblastoma cell lines. Hence, we overexpressed EGFP-luciferase or EGFP-NBPF1 in three different NB cell lines (SH-SY5Y, NLF and SK-N-AS) and 48 h after transfection we analyzed the cell cycle profiles of the positively-transfected cell populations. SH-SY5Y is a NB cell line bearing wild-type *TP53*, whereas NLF and SK-N-AS show a *TP53* loss-of-function mutation due to, respectively, a missense mutation in exon 6 (607G > A) and a homozygous deletion of exons 10 and 11 [12]. Interestingly, EGFP-NBPF1 overexpression arrested the cell cycle in the G1 phase in the two p53-mutant NB cell lines, whereas overexpression of EGFP-NBPF1 in the SH-SY5Y NB cell line, with a wild-type *TP53*, resulted in a marked sub-G1 peak, indicating induction of cell death (Fig. 10). As a control for efficient EGFP-NBPF1 expression, we determined the protein levels of NBPF1 and confirmed that, as expected, only cells transfected with EGFP-NBPF1 expressed NBPF1, whereas cells transfected with EGFP-luciferase did not (Fig. 5).

**Fig. 11:**
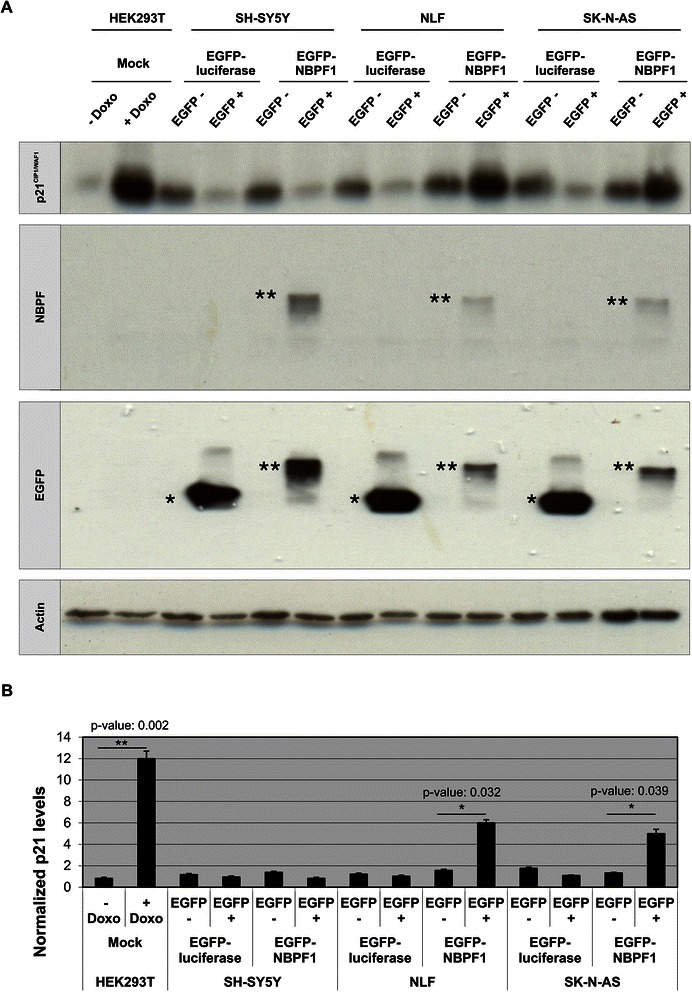
NBPF1 overexpression increases p21^CIP1/WAF1^ protein levels in NB cell lines with mutant p53. (**A**) SH-SY5Y, NLF and SK-N-AS cells were transfected with plasmids encoding either EGFP-luciferase or EGFP-NBPF1. Both EGFP-positive (+) and EGFP-negative populations (−) were isolated by FACS, and the protein levels of p21^*CIP1/WAF1*^ were detected by western blotting (top row). Treatment of HEK293T cells with doxorubicin (+ Doxo) served as a positive control for p21 induction. Detection with the anti-NBPF antibody (sc-82241) showed successful isolation of EGFP-NBPF1 transfected cells (second row). Detection with an anti-EGFP antibody (third row) showed successful isolation of transfected cells that were EGFP-luciferase positive (indicated by *) or EGFP-NBPF1 positive (indicated by **). Actin expression acted as a loading control (bottom row). (**B**) Quantification of p21^CIP1/WAF1^ signals in the blot of (**A**), normalized against actin signals. In addition to the positive control (HEK293T + Doxo), only NB cells with mutant p53 expressing EGFP-NBPF1 showed clear induction of p21. p-values were calculated with one-way ANOVA

**Fig. 12:**
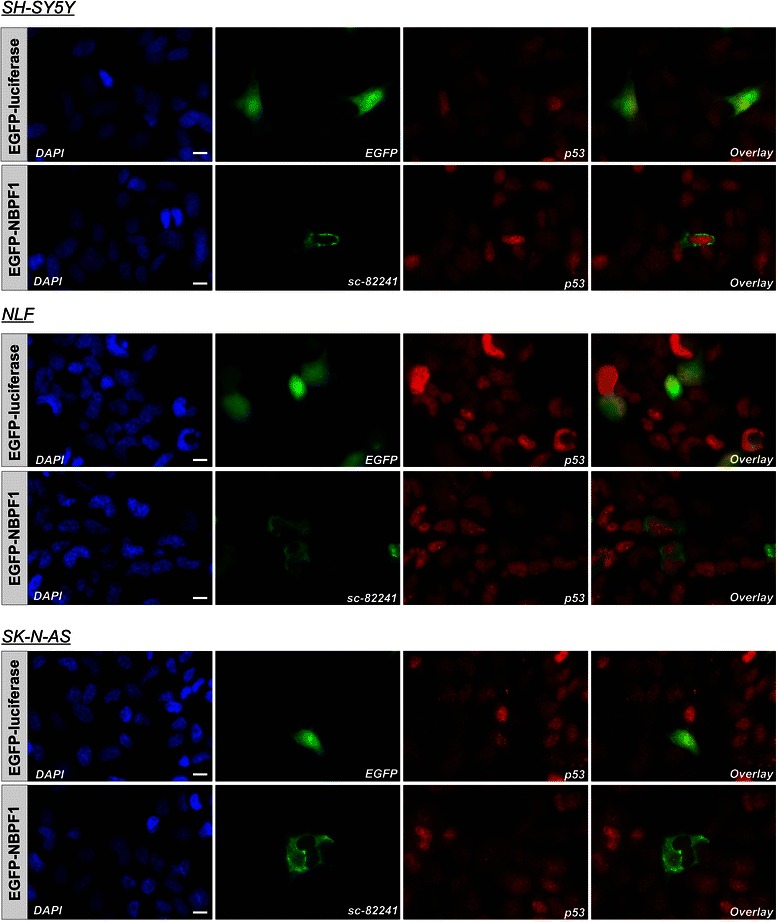
NBPF1 overexpression induces no nuclear accumulation of p53 in NB cell lines. SH-SY5Y, NLF and SK-N-AS cells were transfected with a plasmid encoding either EGFP-luciferase (top panel) or EGFP-NBPF1 (lower panel). Immunofluorescence for p53 revealed no increased accumulation in the nucleus upon EGFP-NBPF1 transfection in either cell line. EGFP-luciferase transfected cells were stained with an anti-EGFP antibody, whereas EGFP-NBPF1 transfected cells were stained with the anti-NBPF antibody sc-82241, both represented in green. Nuclei were counterstained with DAPI. Scale bars: 10 μm

Moreover, analysis of protein lysates of EGFP-positive and EGFP-negative subpopulations from both settings of the three NB cell lines showed that the p21^CIP1/WAF1^ protein levels were upregulated upon NBPF1 overexpression in NLF and SK-N-AS cells, in line with the observed G1 cell cycle block, but not in SH-SY5Y cells (Fig. 11). We also performed immunofluorescence for EGFP, NBPF1 and p53 in order to check whether the overexpression of NBPF1 resulted in nuclear accumulation of p53. However, we did not observe marked nuclear accumulation of p53 upon NBPF1 overexpression in either NB cell line (Fig. 12).

Together, these data indicate that NBPF1 overexpression in NB cell lines with non-functional p53 results in G1 cell cycle arrest and induction of *CDKN1A*, whereas overexpression of NBPF1 in a NB cell line with a wild-type p53 triggers cell death without induction of *CDKN1A*.

### *CDKN1A* expression is not induced upon NBPF1 expression in DLD1Tr21/NBPF1 cells

**Fig. 13:**
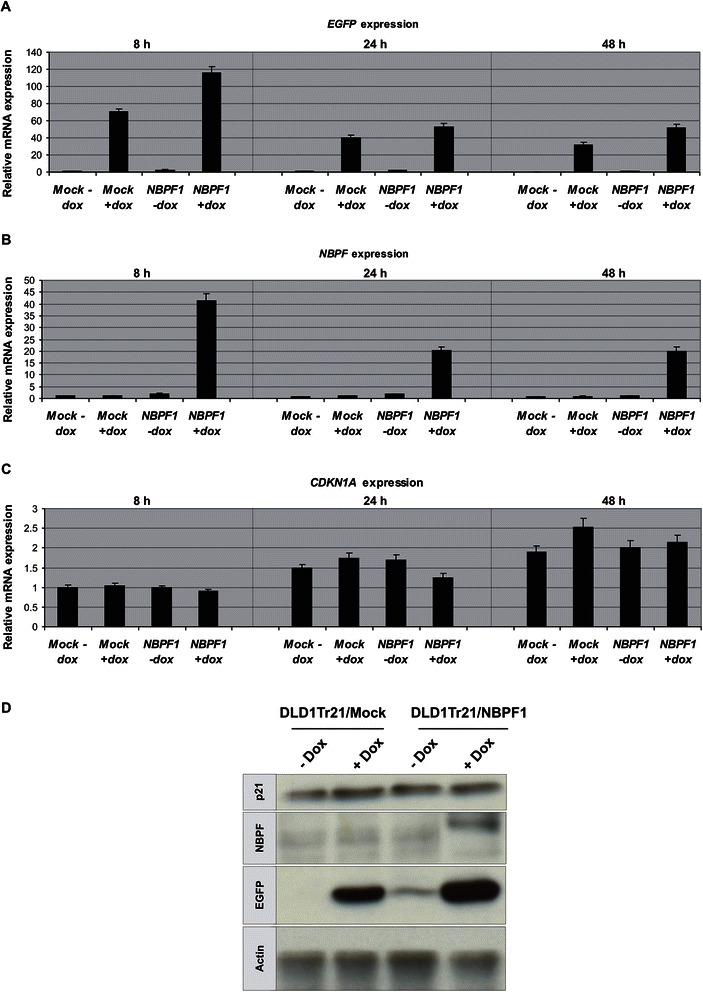
*CDKN1A* expression is not induced upon NBPF1 expression in DLD1Tr21/NBPF1 cells. Expression analysis of selected genes and proteins was executed in DLD1Tr21/Mock and DLD1Tr21/NBPF1 cells, with or without dox treatment. Real-time quantitative RT-PCR measurements are shown for the expression levels of the IRES-driven marker gene *EGFP* (**A**), *NBPF1* (**B**) and *CDKN1A* (**C**). Cells analyzed were kept untreated (− dox) or were dox treated for 8, 24 or 48 h in order to induce NBPF1 expression. *CDKN1A* mRNA induction upon NBPF1 expression was not observed in the dox-induced DLD1Tr21/NBPF1 cells. (**D**) Western blot was performed with lysates of dox-treated and non-treated DLD1Tr21/Mock and DLD1Tr21/NBPF1 cells. Cells were induced for 48 h and immunoblotted with an anti-p21 antibody. p21 protein induction was not observed in dox-induced DLD1Tr21/NBPF1 cells. EGFP detection showed efficient dox-dependent induction in both cell lines. Actin detection was used as a loading control

**Fig. 14:**
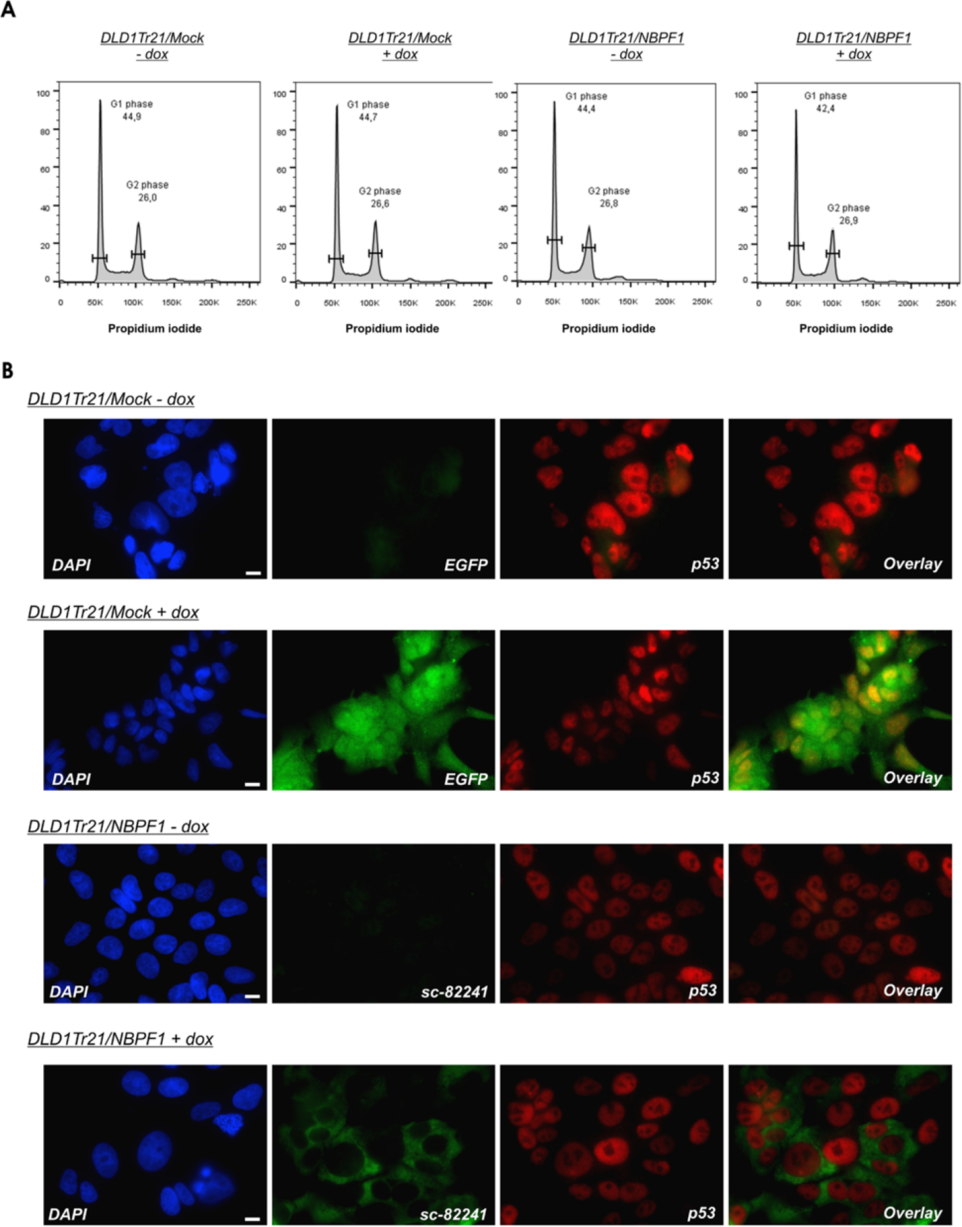
Induced expression of NBPF1 in DLD1Tr21 cells does not result in a G1 cell cycle arrest or nuclear accumulation of p53. (**A**) Cell cycle distribution of DLD1Tr21/Mock and DLD1Tr21/NBPF1 cells in the absence or presence of dox (48 h) shows no G1 arrest upon NBPF1 expression. (**B**) Immunofluorescence for p53 revealed no increased accumulation in the nucleus upon NBPF1 expression. Expression of EGFP or NBPF1 (using antibody sc-82241) is induced upon treatment of the cells with dox for 48 h. Nuclei were visualized with DAPI. Scale bars: 10 μm

We then investigated whether the NBPF1-dependent *CDKN1A* induction could be confirmed in p53-mutant DLD1Tr21/NBPF1 cells, in which we expect that NBPF1 plays a tumor suppressor role [3]. DLD1Tr21/NBPF1 and DLD1Tr21/Mock cells were induced with dox for 8, 24 or 48 h and the expression levels of *CDKN1A*, *EGFP* and *NBPF* transcripts were determined by real-time quantitative RT-PCR. This expression analysis confirmed that DLD1Tr21/NBPF1 and DLD1Tr21/Mock cells expressed EGFP upon dox addition (Fig. 13A), and that NBPF1 expression was induced only in the DLD1Tr21/NBPF1 cell line in the presence of dox (Fig. 13B). However, in this cell line we did not detect any NBPF1-dependent changes in the mRNA level of *CDKN1A* (Fig. 13C). In addition, protein lysates of induced DLD1Tr21/NBPF1 cells showed that also the p21^CIP1/WAF1^ protein levels were not upregulated upon NBPF1 overexpression (Fig. 13D). Moreover, investigation of the different cell cycle phases upon induction of NBPF1 showed no changes in G1 or G2 phases (Fig. 14A), nor did we observe nuclear accumulation of p53 upon NBPF1 induction (Fig. 14B). These data indicate that the induction of *CDKN1A* upon NBPF1 overexpression is cell-type specific, and that the tumor suppressive effect of NBPF1 may act with or without cell cycle arrest.

### The proteome response of DLD1Tr21 cells upon expression of NBPF1

**Fig. 15:**
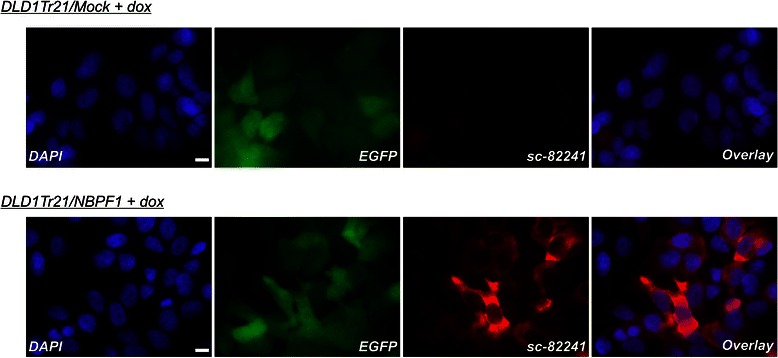
Immunofluorescent analysis of the DLD1Tr21/Mock and DLD1Tr21/NBPF1 cell cultures used for proteomic analysis. Cell cultures were grown for 4 days in standard medium supplemented with dox. The induced DLD1Tr21/Mock cells were post-metabolically labeled with light ^12^C_6_-NHS-propionate, and the induced DLD1Tr21/NBPF1 cells were post-metabolically labeled with heavy ^13^C_6_-NHS-propionate, and then submitted to proteomic analysis as described in Methods. As a control for proper NBPF1 induction, replicate cell cultures were stained for induction of EGFP and NBPF1 expression by immunofluorescent microscopy. Expression of EGFP (shown in green) is induced in both cell lines upon the addition of dox (untreated cultures were fully negative). NBPF1 (antibody sc82241; red channel) was expressed only in DLD1Tr21/NBPF1 cells and only in the presence of dox. Nuclei were visualized with DAPI. Scale bars: 10 μm

To obtain complementary information with respect to the role of NBPF1, we investigated the differences between the proteomes of DLD1Tr21/NBPF1 cells and DLD1Tr21/Mock cells. Both cell types were grown for 4 days in the presence of dox in standard medium. Lysates of the induced DLD1Tr21/Mock cells (Fig. 15; + dox, no NBPF1 expression, only EGFP expression) were digested with endoproteinase Lys-C and post-metabolically labeled with light NHS-^12^C_3_-propionate [17]. On the other hand, lysates of the induced DLD1Tr21/NBPF1 cells (Fig. 15; + dox, NBPF1 and EGFP expression) were labeled with heavy NHS-^13^C_3_-propionate. LC-MS/MS analysis led to the identification of 3613 proteins. A total of 32 proteins showing differential expression was identified at the 99% confidence level [18] (Additional file 5: Table S1). Of these 32 proteins, 19 proteins were upregulated, whereas 13 proteins were downregulated upon NBPF1 expression.

**Fig. 16:**
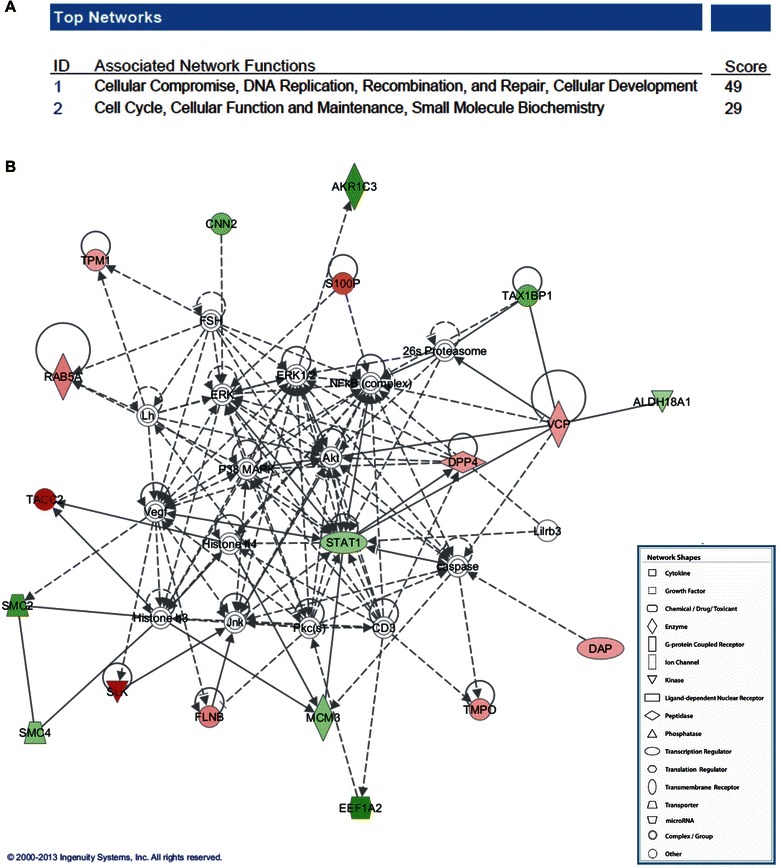
Summary of Ingenuity Pathway Analysis (IPA). The dataset representing the 32 genes with significantly altered expression upon NBPF1 induction in DLD1Tr21 cells was imported into the IPA tool. (**A**) The top two networks obtained from the IPA analysis and their scores. (**B**) Network presentation of the most highly rated network. Solid lines represent a direct interaction between two gene products and dotted lines represent indirect interactions. Red symbols indicate input molecules that are downregulated upon NBPF1 expression, and green symbols represent input molecules that are upregulated upon NBPF1 expression

To investigate the possible biological interactions between the proteins that were differently regulated upon NBPF1 expression, the proteomics data were imported into the Ingenuity Pathway Analysis (IPA) tool. This analysis revealed two networks that are significantly altered upon NBPF1 expression, of which ‘cellular compromise, DNA replication and recombination’ was the highest rated network, with 19 focus molecules and a significance score of 49 (Fig. 16A). The IPA analysis also grouped the genes that were differentially expressed upon NBPF1 expression into a number of biological mechanisms related to dermatological disease, inflammatory disease, inflammatory response, cancer, and endocrine system disorders. Interestingly, 13 of the 14 genes that were differentially expressed and implicated in cancer (p-value ranging from 3.74E–4 to 4.77E–2) were present in the highest rated network (Fig. 16B).

## Discussion

We previously reported evidence for a tumor suppressor role of NBPF1 [3]. However, the molecular mechanisms responsible for its anti-tumor effect were not elucidated. This paper shows that NBPF1 can exert its tumor suppressive effect in different ways: by blocking cell division, inducing cell death, or by modulating a cancer-associated proteome.

Live-cell imaging of the embryonic kidney cell line HEK293T, transfected with an EGFP-NBPF1 expressing construct, showed a temporally fluctuating pattern of EGFP-NBPF1 expression in comparison with control transfected cells continuously expressing EGFP-luciferase. In addition, we did not observe any EGFP-NBPF1 transfected cell that completed a cell cycle, whereas control EGFP-luciferase transfected cells and mock transfected cells divided frequently. Analysis of the different stages of the cell cycle in this cell line showed that overexpression of NBPF1 resulted in a clear G1-arrest in comparison with the controls. In the HEK293T cells, this arrest could be explained by the specific induction of p21^CIP1/WAF1^, seen at both the mRNA and protein levels.

The best known activator of p21^CIP1/WAF1^ is the tumor suppressor p53 [19]. Activation of p21 by p53 has indeed been shown to play a key role in the ability of p53 to induce cell cycle arrest [20]. Therefore, we performed experiments to determine if NBPF1 was acting via p53 induction. First, we performed immunofluorescence for p53 in HEK293T cells and showed an increased nuclear accumulation of p53 upon overexpression of NBPF1, whereas knock-down of p53 did not result in a G1 cell cycle arrest and abolished NBPF1-induced *CDKN1A* expression. Still, the p53 status in HEK293T cells is controversial as these cells express adenoviral proteins that inhibit p53 activity. To clarify this controversy, we stimulated HEK293T and DLD1Tr21 cells with an inducer of DNA double-strand breaks, doxorubicin, which results in p53-dependent upregulation of p21 [21]. Treatment with doxorubicin induced p21 in the HEK293T cells, but not in the DLD1Tr21 cell line with mutated *TP53* (data not shown). Therefore, we concluded that the activity of p53 is only attenuated in HEK293T cells, but not completely inhibited. This leads to transcriptional activation of *CDKN1A* upon doxorubicin treatment, and therefore, the induction of p21 upon overexpression of NBPF1 in HEK293T cells can be considered to be p53-dependent. Moreover, stable knockdown of p53 in HEK293T cells completely abrogated the NBPF1-induced cell cycle block and *CDKN1A* upregulation, thereby confirming that NBPF1-induced cell cycle arrest is p53-dependent in HEK293T cells.

Nuclear translocation of p53 indicates its activation [22], and NBPF1 seems to act in HEK293T cells via increasing the nuclear expression of p53. However, the mechanism by which NBPF1 can modulate p53 remains unknown. As NBPF1 proteins are located in the cytoplasm, whereas active p53 is in the nucleus, a direct interaction between NBPF1 and p53 seems unlikely. However, MDM2, the main inhibitor of p53 activity, is located in the cytoplasm, and NBPF1 might interact with MDM2, thereby preventing the negative effects of MDM2 on p53. To test this hypothesis, we performed co-immunoprecipitation experiments after overexpression of both NBPF1 and MDM2 in HEK293T cells. Although we could demonstrate a minor interaction between NBPF1 and MDM2 (data not shown), we believe that this interaction is of insufficient extent to explain the observed NBPF1-driven nuclear accumulation of p53. On the other hand, it is possible that NBPF1 promotes the nuclear localization of p53 via a posttranslational modification, such as phosphorylation or acetylation [23], but additional experiments are required to elucidate this.

The human *NBPF1* gene was originally identified in a NB patient [1-3], and we reported previously that expression of *NBPF1* mRNA is significantly decreased in NB cell lines with loss of heterozygosity for 1p36 compared to cell lines with a normal 1p36 locus [3]. Therefore, we investigated the role of NBPF1 in several NB cell lines, both with mutant or wild-type *TP53* alleles. Overexpression of NBPF1 in NLF and SK-N-AS resulted in a G1 cell cycle arrest and in *CDKN1A* upregulation. NLF and SK-N-AS bear loss-of-function mutations of *TP53*, namely, a missense mutation in the *TP53* exon 6 (607G > A) of NLF cells, and a homozygous deletion of *TP53* exons 10 and 11 of SK-N-AS cells [12]. These results indicate that the G1 cell cycle arrest is p53-independent or that these mutant p53 proteins can still induce p21. Stable knockdown of p53 in these NB cell lines is required to confirm that NBPF1-induced cell cycle arrest is p53-independent in these NB cell lines. Surprisingly, overexpression of NBPF1 in the SH-SY5Y cell line, expressing wild-type p53, did not result in a G1 cell cycle arrest, but led to the appearance of a sub-G1 peak, indicative of induced cell death. Therefore, we hypothesize that the growth-inhibitory effect upon NBPF1 overexpression is p21-dependent, and that overexpression of NBPF1 in the SH-SY5Y cell line does not result in G1 arrest due to lack of p21 induction.

The observed arrest of the cell cycle by overexpression of NBPF1 is in line with the apparent expression of NBPF family members in the non-proliferating suprabasal layers of squamous stratified tissues, whereas the basal layers with proliferating cells are essentially NBPF-negative. We demonstrated this by immunohistochemistry using a polyclonal antibody after having clearly demonstrated its specificity for NBPF proteins. This commercial antibody might therefore be used in future studies on NBPF expression in human tumor samples.

We could not confirm NBPF1-dependent *CDKN1A* upregulation in DLD1Tr21/NBPF1 colon cancer cells. Our proteome analysis of DLD1 cells with induced NBPF1 expression indicates that the absence of a cell cycle arrest in these cells might be due to the upregulation of several cell-cycle promoting genes, such as *EEF1A2* and *MCM3* [24, 25]. Whether these genes are directly upregulated by NBPF1 or whether their upregulation represents compensatory effects counteracting NBPF1 function in DLD1 cells needs further investigation. In any case, these data indicate that the proposed tumor suppressive properties of NBPF1 might be very versatile and cell-specific.

Despite the lack of *CDKN1A* upregulation in DLD1 cells with inducible NBPF1 expression, these cells show a marked decrease of clonal growth in a soft agar assay upon NBPF1 induction [3]. Anchorage-independent growth of cells is one of the hallmarks of cellular transformation and is regarded as a critical step during metastasis [26]. Intriguingly, VCP (valosin-containing protein), which is associated with anti-apoptotic functions and metastasis via activation of the NF-κB signaling pathway [27], was significantly downregulated upon NBPF1 expression in DLD1 cells. Moreover, NBPF1 induction decreased the expression of S100P, the expression of which is associated with drug resistance, metastasis, and poor clinical outcome in many different cancers such as colon, breast and prostate cancer [28]. Also, overexpression of S100P in PC3 prostate cancer cells promotes anchorage-independent growth in soft agar [29].

Both overexpression and knockdown strategies are commonly used to scrutinize gene functions. Thus, knockdown of *NBPF1* would have been a complementary approach to the experiments described here. We used several approaches and numerous attempts to knock down the expression of the *NBPF1* gene or the *NBPF* gene family in human cells (Table 2). On the one hand, we aimed at silencing specifically the *NBPF1* gene. On the other hand, we tried to silence the *NBPF* gene family as a whole because downregulation of only *NBPF1* might lead to compensation by *NBPF* paralogs having overlapping functions and therefore obscure the phenotype. However, none of the many siRNAs and shRNAs tested showed an acceptable knockdown level for silencing endogenous NBPF, which has prevented us so far from proceeding with functional studies.

**Table 2:** Overview of shRNAs and siRNAs used in this study with the aim to silence the expression level of *NBPF* family members

Knockdown type	#	Target seq (5' to 3')	Targets only *NBPF1*		Exon type
			No	Yes	
shRNA_19-mer	1	CCUCUUCUGCCACAAACGUUU	X		1
	2	GGAAUGUGCCAUCACUUGU	X		5
	3	GGAAGUCCCUGAGGACUCA	X		6
	4	GAAGAAGAUCAACGAAGAA		X	12
	5	CUUACUUUGAUGGGAACAA		X	14
shRNA_29-mer	6	UACCUCUUCUGCCACAAACGUCAGCAUGGU	X		1
	7	AUGUCAGGAUGCUGUAAACAUUCUCCCAG		X	5
	8	AACAUCAACAUCACCUUUGAGGAAGACAA		X	5
	9	UGACUCCAACCAGCCUCACAAGAACAUCA	X		5
	10	GGAGAAGACGGUGUACCAGCCUCACAAGA	X		5
	11	ACCAGUCUUACAGCGGCACAUUUCACUCA		X	8
	12	CCUGACUCAUUCCAGCACUACAGAAGUGU	X		14
	13	AGCUGGCAGAGAACAAACAGCAGUUCAGA	X		1
	14	AUGCUGAGGAAUGAGCGACAGUUCAAGGA	X		2
	15	UAAGGGAGAAGUUACGGGAAGGGAGAGAU	X		3
	16	CUUCCUGGCCAACCAGCAGAACAAAUACA	X		1
siRNA pool	17	GAAUCGUACUGCUAAGAAU		X	5' UTR
		ACAGAGACAGACAAAGAUA	X		5' UTR
		GAUGGCCAAUCUUUCCUAA	X		5' UTR
		GCAAGUCCAGGUCAUACUG	X		5' UTR

Given are the target sequence, the predicted specificity (NBPF1-specific or broad NBPF specificity) and location in exons (for exon type, see ref. [4]) or 5’UTR. shRNAs 1 to 5 represent shRNAs with a stem length of 19 bps, whereas shRNAs 6 to 16 represent shRNAs with a stem length of 29 bps. For shRNAs 1 to 8, the expression cassettes of the shRNAs were created by us by a double PCR-approach, whereas shRNAs 9 to 16 are HuSH retroviral silencing vectors from Origene (Origene Technologies, Rockville, MD, USA). siRNA pool 17 represents an On-Target plus siRNA pool from Dharmacon (Thermo Fisher Scientific, Lafayette, CO, USA)

## Conclusions

We have shown that NBPF1 can act as a tumor suppressor by inhibiting the cell cycle, by cell death induction or by expression modulation of various proteins implicated in cancer. This may occur via either p53-dependent or p53-independent mechanisms, depending on the cell type. Therefore, disruption of the *NBPF1* gene in neuroblastoma patients, and possibly in other cancers as well, can result in several dysregulations of cellular activity and thereby contribute to the initiation or progression of cancer.

This study is among the first molecular analyses of the tumor suppressor functions of NBPF proteins. Further progress in the functional characterization of these intricate proteins, which probably have important roles in human cancer, should help us to improve human cancer diagnosis and prognosis, and to develop more effective therapeutic strategies.

## Additional files

Additional file 1: Movie 1. Fluorescence time lapse microscopy of HEK293T cells expressing EGFP-luciferase. EGFP-luciferase expression was followed for 15 h with images captured every 10 min. Fluorescence levels are represented by pseudocolors, where blue indicates low EGFP levels and yellow indicates high EGFP levels. EGFP-luciferase expression remained constant during time lapse microscopy. Cells regularly underwent mitosis.
Additional file 2: Movie 2. Fluorescence time lapse microscopy of HEK293T cells expressing EGFP-NBPF1. EGFP-NBPF1 expression was followed for 15 h with images captured every 10 min. Fluorescence levels are represented by pseudocolors, where blue indicates low EGFP levels and yellow indicates high EGFP levels. EGFP-NBPF1 expression decreased rapidly or EGFP-negative cells became modestly EGFP-positive during the experiment. EGFP-NPBP1 expressing mitotic cells successfully completing mitosis were never observed.
Additional file 3: Movie 3. Fluorescence time lapse microscopy of HEK293T cells stimulated with aphidicolin and expressing EGFP-NBPF1. EGFP-NBPF1 expression was followed for 15 h with images captured every 10 min. Fluorescence levels are represented by pseudocolors, where blue indicates low EGFP levels and yellow indicates high EGFP levels. EGFP-NBPF1 expression increased during the experiment and protein aggregates were formed.
Additional file 4: Movie 4. Fluorescence time lapse microscopy of HEK293T cells stimulated with aphidicolin and expressing EGFP-luciferase. EGFP-luciferase expression was followed for 15 h with images captured every 10 min. Fluorescence levels are represented by pseudocolors, where blue indicates low EGFP levels and yellow indicates high EGFP levels. EGFP-luciferase expression remained constant during time lapse microscopy.
Additional file 5: Table S1. List of differential protein candidates upon NBPF1 expression. 32 proteins showing differential expression were identified at the 99 % confidence interval. Accession number, peptide number, gene name, protein description, fold change and peptide sequences are shown in the table. Proteins that are up or down regulated upon NBPF1 expression are shown in green and red, respectively.

## Abbreviations

Dox: Doxycycline hydrochloride
Doxo: Doxorubicine hydrochloride
EGFP: Enhanced green fluorescent protein
FACS: Fluorescence-activated cell sorting
NB: Neuroblastoma
qRT-PCR: Quantitative reverse transcription polymerase chain reaction.
